# Interaction of Epstein-Barr virus genes with human gastric carcinoma transcriptome

**DOI:** 10.18632/oncotarget.16417

**Published:** 2017-03-21

**Authors:** Ruiyuan Zhang, Michael J. Strong, Melody Baddoo, Zhen Lin, Yu-Ping Wang, Erik K. Flemington, Yao-Zhong Liu

**Affiliations:** ^1^ Department of Global Biostatistics and Data Science, Tulane University School of Public Health and Tropical Medicine, New Orleans, LA, USA; ^2^ Department of Pathology and Laboratory Medicine, Tulane Cancer Center, Tulane University Health Sciences Center, New Orleans, LA, USA; ^3^ Department of Biomedical Engineering, Tulane University School of Science and Engineering, New Orleans, LA, USA

**Keywords:** EBV, RNA-seq, gastric carcinoma

## Abstract

Gastric carcinoma (GC) is a leading cause of mortality. 10% of GC cases are related with EBV (Epstein-Barr virus) infection. The detailed mechanistic roles EBV genes play and especially the interaction between the viral genes and human genes in GC remain unclear. In this study, raw fastq data from 285 GC samples were downloaded from TCGA (The Cancer Genome Atlas), including 25 EBV positive (EBV+) GC samples and 260 EBV negative (EBV−) GC samples. RNA-seq based expression data were generated for both human genes (among all the samples) and for the EBV genes (among the 25 EBV+ samples). Bioinformatics analyses were performed to identify differentially expressed (DEx) human genes and DEx KEGG pathways in EBV+ vs. EBV− samples and co-expressed human gene modules and hub genes among the DEx genes. Within the EBV+ samples, analyses were conducted to find correlation between EBV gene expression and the human gene expression modules, between EBV gene expression and the human hub genes, and between EBV gene expression and the DEx human pathways. EBV genes LMP-1, BALF1 and BALF2 were found to have significant correlation with human hub genes, CNTD2 and VANGL2. EBV genes BALF4 and BALF5 were found to correlate with human pathways, including Jak-STAT signaling and Phosphatidylinositol Signaling System. Our study has revealed the coordinated expression patterns between EBV and human GC transcriptome and identified several key EBV genes that may play an important role in EBV+ GC pathogenesis through their interactions with human genes and pathways.

## INTRODUCTION

Gastric carcinoma (GC) is the fifth most common cancer and the third leading cause of mortality from cancers in the world [1]. Infections and other environmental agents, e.g., tobacco and alcohol and salted preserved food intake, were found to contribute to GC pathogenesis [2, 3]. In particular, EBV (Epstein–Barr virus) infection is one of the most important factors that cause GC [2, 4].

EBV is a cancer-related virus, which was observed to cause various human cancers in epithelial cells, lymphocytes and mesenchymal cells [5–7] and was found to account for 10% of all cases of GC [2, 4]. GC that was related to EBV infection shows a distinct molecular character compared to GC caused by other factors [4]. A recent study [4] in TCGA (The Cancer Genome Atlas) characterized GC into 4 subtypes: EBV−positive (EBV+), Microsatellite instability (MSI), genomically stable, and chromosomal instable. The EBV+ subtype was shown to have extensive DNA promoter hypermethylation [4].

Previous studies revealed that EBV+ GC was shown to lose expression of three critical tumor suppressor gene products, CDH1 (E-cadherin), p73, and CDKN2A (p16) [8–11]. Other studies found that EBV−related GC is a subset of CpG island methylator phenotype (CIMP) cancers [10, 12–14]. However, the specific roles EBV plays at the genomic and molecular level and particularly the interaction between the viral genes and human genes in GC pathogenesis remain unclear. Our study is aimed to fill this gap of knowledge by correlating EBV gene expression with human gene expression in GC cells and identify those EBV genes that may account for human gene expression variation at the whole transcriptome scale.

Our analysis started with differential expression (DEx) analysis that identified differentially expressed (DEx) genes (DEGs) and DEx pathways in EBV infected (EBV+) vs. EBV free (EBV−) human GC samples. Those identified DEGs and DEx pathways represent a subset of the human GC transcriptome that is relevant to EBV infection. Therefore, we focused on these DEGs and DEx pathways to find those human genes/pathways that interact with EBV.

Due to the large number of genes/pathways involved, to avoid false positives due to multiple testing, we used a number of dimension reduction tools, including MEGENA [15] to extract hub genes and gene modules from the DEGs, and PCA (principal component analysis) to extract the first PC (principal components) of gene modules and DEx pathways. We then correlated the EBV genes with these extracted features through both a univariate (Pearson correlation) and a multivariate approach (sparse canonical correlation analysis, abbreviated as sCCA). Our analysis narrowed down the EBV gene list to a few EBV genes paired with a limited number of human genes/pathways with significant correlation. These identified EBV genes and their “partners” of human genes/pathways may represent key interactive players in EBV−related GC pathogenesis, whose importance is supported by some previous research evidences.

## RESULTS

### Sample selection

A total of 20 EBV− samples were selected. In Supplementary Table 11, we list the selected EBV− samples and their distance to the EBV+ samples. As shown in Supplementary Table 11, for each EBV+ sample, the distance to the selected EBV− sample is much smaller than its average distance to all the 260 EBV− samples. Also shown is the fact that several EBV+ samples share a same EBV− sample as their closest EBV− counterpart, thus the number of EBV− samples is only 20 while the number of the EBV+ samples is 25.

### Data cleaning

**Figure 1:**
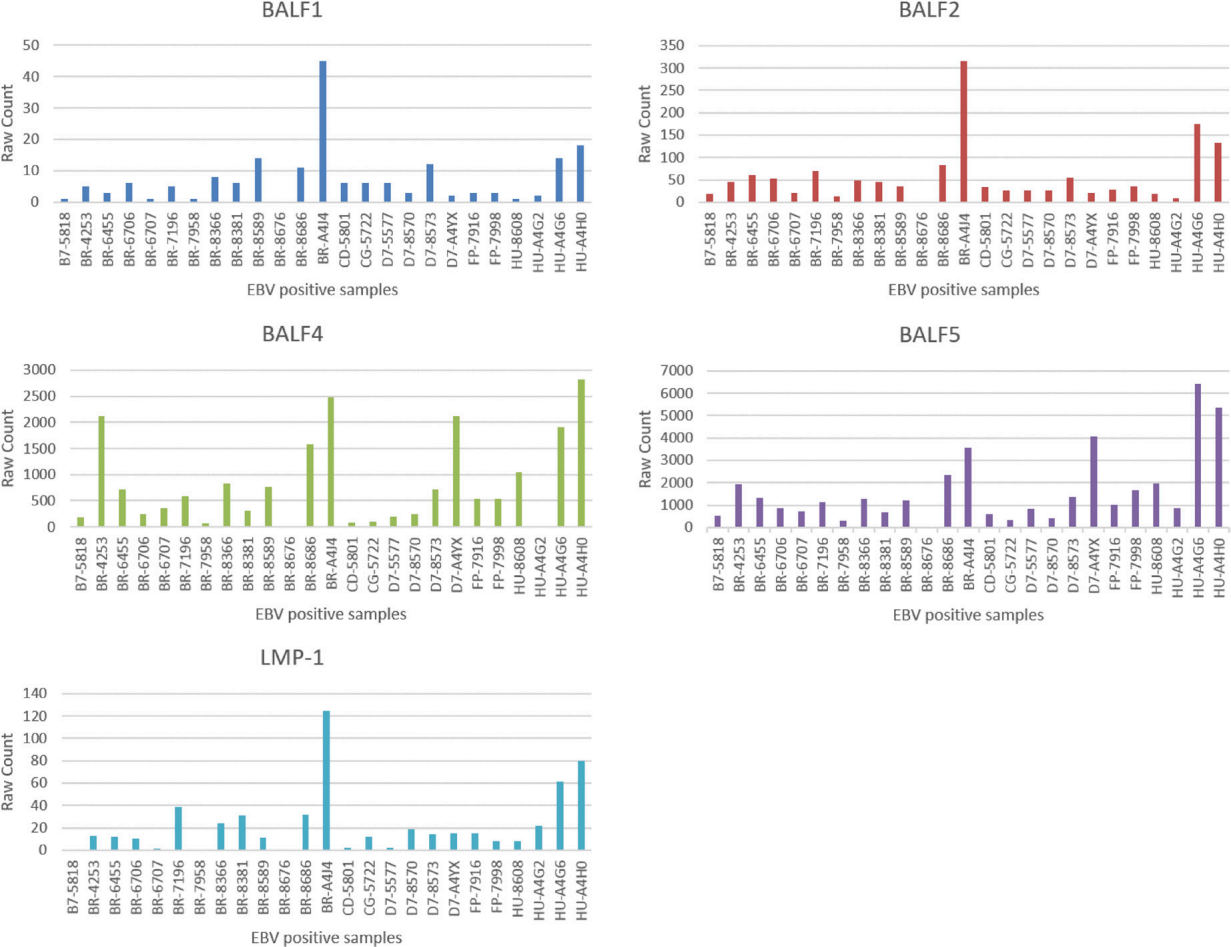
Read counts of 5 EBV genes

Nineteen out of 88 EBV genes remained after data cleaning, where the genes that have a 0-count in greater than 5 EBV+ samples were removed. These 19 EBV genes are: A73, BALF1, BALF2, BALF3, BALF4, BALF5, BARF0, BARF1, BNRF1, BRLF1, BZLF1, LF1, LF2, LF3, LMP-1, LMP-2A, LMP-2B, Qp-EBNA1, and RPMS1. The raw counts for these 19 EBV genes across the 25 EBV+ samples are shown in Figure 1.

### Differential expression analysis in EBV+ vs. EBV− samples

A total of 939 genes were found to be differentially expressed at a significance level of Bonferroni corrected *p* value < 0.05, including 189 genes upregulated and 750 downregulated in EBV+ vs. EBV− samples. The gene symbols and direction of regulation (up or downregulation) for these genes is included in Supplementary Table 2.

### Pathway analysis

At the significance level of FDR adjusted *p* < 0.05, we identified 29 KEGG pathways that are DEx in EBV+ vs. EBV− samples (Table 1). All of the 29 DEx pathways are upregulated in EBV+ vs. EBV− samples.

**Table 1:** Differentially expressed pathways in EBV+ vs. EBV− samples

Pathways	*P* value	FDR
hsa04612 Antigen processing and presentation	< 0.001	< 0.001
hsa04650 Natural killer cell mediated cytotoxicity	< 0.001	< 0.001
hsa03040 Spliceosome	< 0.001	< 0.001
hsa04380 Osteoclast differentiation	< 0.001	< 0.001
hsa04120 Ubiquitin mediated proteolysis	< 0.001	0.001
hsa04110 Cell cycle	< 0.001	0.001
hsa04620 Toll-like receptor signaling pathway	< 0.001	0.001
hsa03030 DNA replication	< 0.001	0.001
hsa04660 T cell receptor signaling pathway	< 0.001	0.001
hsa03010 Ribosome	< 0.001	0.001
hsa04142 Lysosome	< 0.001	0.001
hsa03420 Nucleotide excision repair	< 0.001	0.002
hsa04141 Protein processing in endoplasmic reticulum	< 0.001	0.002
hsa04630 Jak-STAT signaling pathway	0.001	0.008
hsa03013 RNA transport	0.001	0.008
hsa03050 Proteasome	0.001	0.008
hsa04210 Apoptosis	0.001	0.01
hsa04672 Intestinal immune network for IgA production	0.001	0.01
hsa03008 Ribosome biogenesis in eukaryotes	0.001	0.011
hsa04623 Cytosolic DNA-sensing pathway	0.001	0.011
hsa04666 Fc gamma R-mediated phagocytosis	0.001	0.011
hsa03430 Mismatch repair	0.001	0.011
hsa00240 Pyrimidine metabolism	0.002	0.014
hsa04062 Chemokine signaling pathway	0.002	0.014
hsa00970 Aminoacyl-tRNA biosynthesis	0.002	0.014
hsa04622 RIG-I-like receptor signaling pathway	0.003	0.02
hsa04115 p53 signaling pathway	0.004	0.024
hsa04070 Phosphatidylinositol signaling system	0.005	0.028
hsa03018 RNA degradation	0.006	0.035

### Gene co-expression analysis

MEGENA identified 27 modules and 91 hub genes in the 939 DEx human genes. Note that since one gene may be included in different modules, the total number of genes in modules may be greater than 939. Supplementary Table 3 shows the modules and the number of genes contained in each module. Supplementary Table 4 shows the name of the 91 hub genes.

### PCA of modules and pathways

We used first PC (PC1) of each module's expression data to represent the module. The percent of variation explained by the PC1 of each module ranges from 44.5% to 74.5% with a mean of 54.8% and a standard deviation of 7.8%. The detailed percentage of variation explained by PC1 of each module is listed in Supplementary Table 5.

PCA of the 29 significant pathways (Table 1) was also performed and the variation explained by the PC1 ranges from 18.4% to 63.4%, with a mean of 32.6% and a standard deviation of 8.6%. The detailed percentage of variation explained by PC1 of each pathway is listed in Supplementary Table 6.

### Pearson correlation analysis of DEx human genes with EBV genes

Seven EBV genes showed significant correlation with the PC1s of 12 modules at the significant level of FDR adjusted *p* value < 0.1. The result is shown in Table 2. Among all the significant EBV−module pairs, the strongest correlation was observed for BALF1 vs. module 12 with a correlation coefficient of 0.662. The two EBV genes, LMP-1 and BALF2, appeared to be correlated with most of the modules. LMP-1 was correlated with six and BALF2 with four modules.

**Table 2:** Human gene modules that correlate with EBV genes in Pearson correlation analysis

EBV genes	Human gene modules	Correlation coefficients
BALF1	12	0.662
BALF2	2	−0.587
BALF2	21	−0.575
BALF2	8	0.595
BALF2	18	0.584
BALF5	26	0.532
BARF1	17	−0.608
BNRF1	12	0.640
BRLF1	1	0.607
LMP-1	15	−0.633
LMP-1	12	0.615
LMP-1	24	0.607
LMP-1	5	0.602
LMP-1	20	0.592
LMP-1	17	−0.569

Three EBV genes showed significant correlation (FDR < 0.1) with 4 hub genes (Table 3). The strongest correlation was observed between the EBV gene LMP-1 and the human gene C1orf115, with a correlation coefficient of 0.754.

**Table 3:** Human hub genes that correlate with EBV in Pearson correlation analysis

EBV genes	Hub genes	Modules the hub gene belongs to	Correlation coefficients
BALF1	CNTD2	12	0.729
LMP-1	VANGL2	12	0.705
LMP-1	C1orf115	5, 20	0.754
LMP-2B	CISD1	6	0.675

Two EBV genes showed significant correlation with PC1 of four pathways at the significance level of FDR-adjusted *p* value < 0.1 (Table 4). The EBV gene, BALF4, showed significant correlation with 4 pathways. The strongest correlation was for BALF4 with the pathway “Phosphatidylinositol signaling system (HSA04070)” with a correlation coefficient of 0.709.

**Table 4:** Human gene pathways that correlate with EBV in Pearson correlation analysis

EBV genes	Pathway names	Correlation coefficient
BALF4	Apoptosis (HSA04210)	0.702
BALF4	Jak-STAT signaling pathway (HSA04630)	0.690
BALF4	Lysosome (HAS04142)	−0.618
BALF4	Phosphatidylinositol signaling system (HSA04070)	0.709
BALF5	Apoptosis (HSA04210	0.564

### sCCA of human genes with EBV genes

Twenty-two human gene modules achieved significant correlation with the count matrix for 19 EBV genes at the significance level of FDR < 0.1 (Table 5). The essential EBV genes that were accountable for the canonical correlation are LMP-1, BALF1, BALF2, BARF1, BNRF1, LF1, and BZLF1.

**Table 5:** Human gene modules that correlate with EBV genes in sCCA

Human gene modules	sCCA correlation coefficients	FDR	Essential EBV genes
1	0.836	0.078	BRLF1, LMP-1,
2	0.808	0.022	BALF2, LMP-1 LF1,
5	0.828	0.008	LMP-1, BALF1, BALF2, BARF1,
6	0.936	0	LF2
7	0.743	0.012	BALF1, BALF2, BNRF1, LMP-1, LMP-2B
8	0.742	0.050	BALF2
9	0.739	0.058	LMP-1, BALF2, BALF1, BNRF1, BARF0, BZLF1, LF1, BARF1 LMP-2A
10	0.712	0.079	BALF4,
11	0.749	0.078	BALF1, BALF2
12	0.821	0.079	BALF1, BNRF1
14	0.782	0.032	BALF5
15	0.774	0.012	LMP-1
17	0.837	0.078	BARF1
18	0.847	0.012	BALF2, LMP-1, LF1
20	0.869	0.003	LMP-1, BALF1, BARF1, BALF2, BNRF1, BZLF1, BRLF1, LF1,
21	0.742	0.032	BALF2, LMP-1
23	0.782	0.008	BALF5
24	0.762	0.050	LMP-1
25	0.759	0.012	LF1, BALF2,
26	0.782	0.012	BALF5
27	0.706	0.068	BALF2, LMP-2B

The canonical correlation between the human hub gene count matrix and the EBV gene count matrix is 0.806 (*p* = 0.010). The essential EBV genes accountable for the canonical correlation are LMP-1, BALF1, BALF2, BNRF1, and BRLF1, and the essential human genes accountable for the sCCA correlation are VANGL2, C1orf115, CNTD2, KCNJ12, NDNF, SYT1, AGAP11, OCA2, UPK1B, WASF3, TMEM220, KCNK15, MAPK8IP1, RP11-78F17.1, SRPX, and PLA2R1.

All DEx pathways achieved significant canonical correlation with the EBV gene count matrix (FDR < 0.1). The top significant ones (with an FDR of < 0.001) are shown in Table 6, which involve 14 pathways. As shown in the table, BALF4 (appearing 13 times) and BALF5 (appearing 14 times) are the essential EBV genes for canonical correlation with most of the pathways.

**Table 6:** Human gene pathways that correlate with EBV genes in sCCA

Pathways	KEGG ID	Canonical correlation coefficients	FDR	Essential EBV genes
Natural killer cell mediated cytotoxicity	hsa04650	0.86	3e-4	BALF4
Spliceosome	hsa03040	0.87	8e-4	BALF4
Osteoclast differentiation	hsa04380	0.95	3e-4	BALF4
Ubiquitin mediated proteolysis	hsa04120	0.88	8e-4	BALF4, BALF5
Cell cycle	hsa04110	0.91	< 3e-4	BALF5
Toll-like receptor signaling pathway	hsa04620	0.95	< 3e-4	BALF5, LF2, BALF4, A73
Lysosome	hsa04142	0.90	6e-4	BALF4, BALF5
Jak-STAT signaling pathway	hsa04630	0.86	< 3e-4	BALF4
RNA transport	hsa03013	0.93	< 3e-4	BALF5, BALF4
Apoptosis	hsa04210	0.94	< 3e-4	BALF5
Cytosolic DNA-sensing pathway	hsa04623	0.89	8e-4	BALF4, BALF5
Fc gamma R-mediated phagocytosis	hsa04666	0.93	8e-4	BALF5, BALF4, A73
Chemokine signaling pathway	hsa04062	0.94	3e-4	BALF5, BALF4
Phosphatidylinositol signaling system	hsa04070	0.89	3e-4	BALF4

### Result summary for EBV genes

An overall summarization of important EBV genes is presented in Table 7.

**Table 7:** Summary of important EBV genes

Important EBV genes	Correlated with human genes in Pearson correlation analysis			Correlated with human genes in sCCA		
	Number of modules	Hub genes	Number of pathways	Number of modules	Hub genes	Number of pathways
LMP-1	5 modules	C1orf115, VANGL2	-	10 modules	Yes	-
BALF2	4 modules	-	-	10 modules	Yes	-
BALF1	-	CNTD2	-	6 modules	Yes	-
BALF4	-	-	4 pathways	1 module	-	12 pathways
BALF5	1 module	-	1 pathway	3 module	-	9 pathways

*LMP-1*: It achieved significant correlation with 6 human gene modules (modules 5, 12, 15, 17, 20, 24) by Pearson correlation analysis (FDR < 0.1). It also achieved significant correlation with 2 human hub genes, C1orf115 (correlation coefficient = 0.754, FDR < 1e-6) and VANGL2 (correlation coefficient = 0.705, FDR = 0.043).

*BALF2*: It significantly correlates with 4 human modules (modules 2, 8, 18 and 21) from Pearson correlation analysis (FDR < 0.1). It is the essential gene in canonical correlation with 11 human gene modules, including modules 2, 5, 7, 8, 9, 11, 18, 20, 21, 25, 27. It was also the essential EBV gene in canonical correlation with human hub genes.

*BALF1*: It achieved significant correlation with one human hub gene, CNTD2, in Pearson correlation analysis with a correlation coefficient of 0.729 (FDR = 0.046). It is the essential EBV gene in canonical correlation with 6 human gene modules, including module 5, 7, 9, 11, 12 and 20. It is essential gene in the canonical correlation with the human hub genes.

*BALF4*: It achieved significant correlation with 4 human pathways (Lysosome, Jak-STAT signaling pathway, Apoptosis, Phosphatidylinositol signaling system) from Pearson correlation analysis, with FDR values of 0.072, 0.008, 0.017 and 0.006, respectively. It is the essential EBV gene in canonical correlation with human gene module 10. It is the essential EBV gene in the canonical correlation with almost all (12 of) the 14 pathways shown in Table 6.

*BALF5*: It is the essential EBV gene in canonical correlation with 3 human gene modules (14, 23 and 26). It is the essential EBV gene in sCCA correlation with 9 human gene pathways.

### Important human gene modules

Shown in Table 8 are human gene modules that were significantly correlated with EBV genes in both Pearson correlation analysis and sCCA.

**Table 8:** Human gene modules that correlate with EBV genes

Human gene modules	Pearson correlation coefficient	sCCA correlation coefficient
12	0.662	0.821
15	0.633	0.774
17	0.608	0.837
1	0.607	0.836
24	0.607	0.762
5	0.602	0.828
8	0.595	0.742
20	0.592	0.869
2	0.587	0.808
18	0.584	0.847
21	0.575	0.742
26	0.532	0.782

These gene modules were annotated in DAVID [16, 17]. The significant annotation results, including those for module 5 and 12, are shown in Supplementary Tables 7, 8. Module 5 contains terms related to membrane, transmembrane and ion channel. Module 12 contains terms related to calcium ion binding (GO:0005509) and Ghrelin Pathway. Interestingly the latter pathway was found to promote gastrointestinal and pancreatic malignancy [18–20].

**Figure 2:**
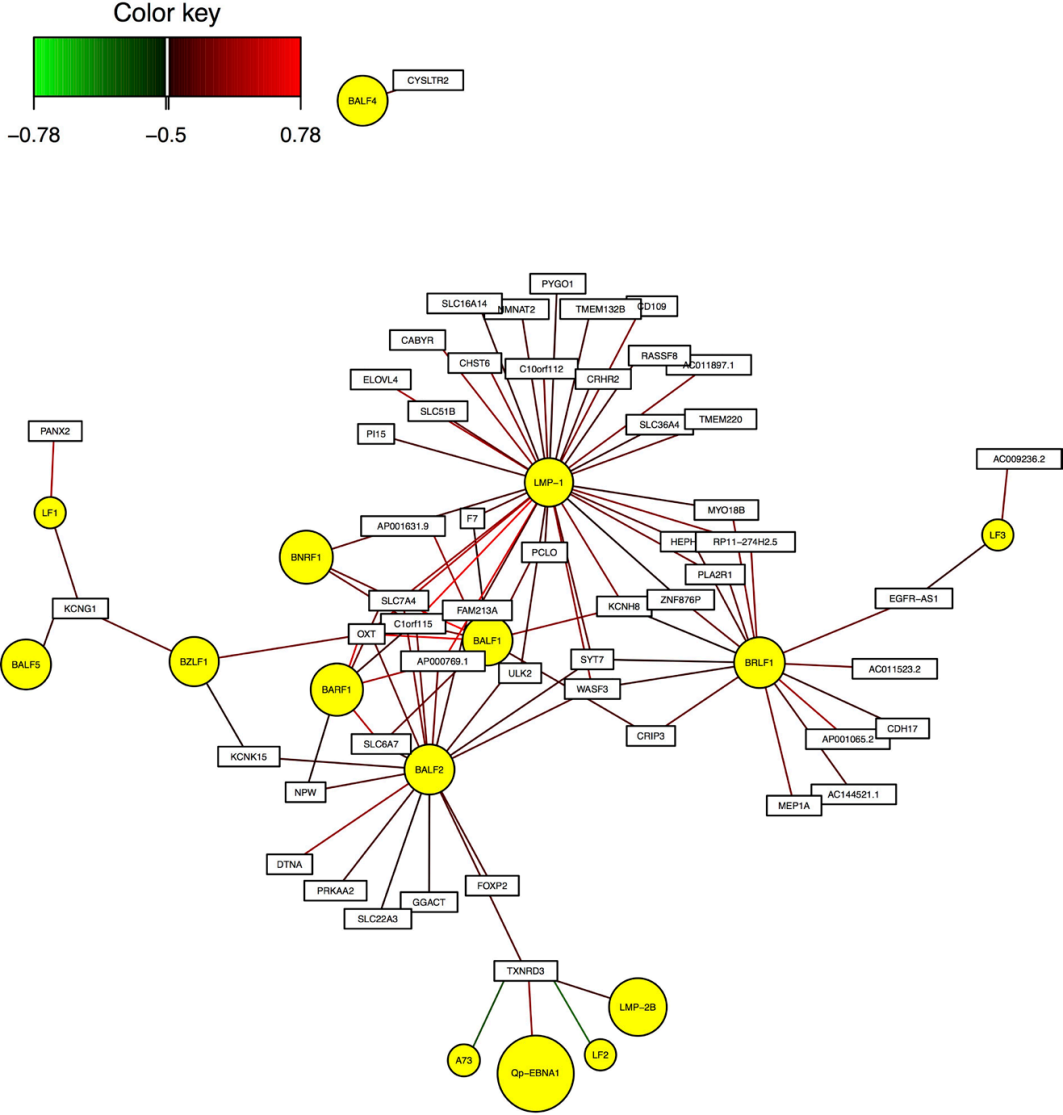
Interaction network between EBV genes and human module 5

As an example, Figure 2 shows the interaction network between module 5 and EBV genes. As shown in the figure, LMP-1, BALF1, BALF2 are located in the center, suggesting that they have high correlation with most of the human genes in these modules.

### Important human hub genes

**Figure 3:**
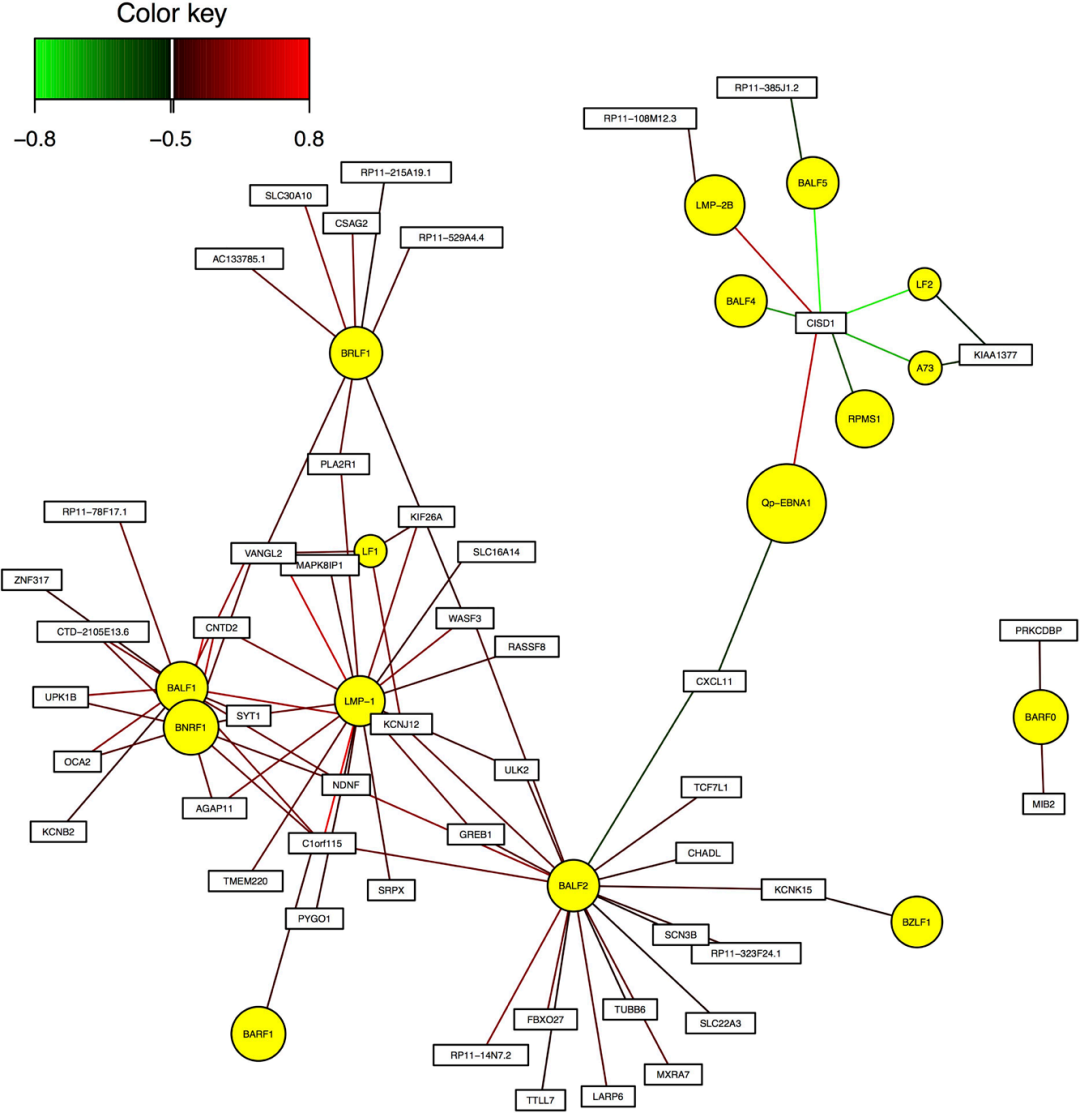
Interaction network between EBV genes and human hub genes

These genes are C1orf115, CNTD2, and VANGL2. They achieved significant correlation with EBV genes BALF1 and LMP-1 in Pearson correlation analysis, and were also identified as essential genes in sCCA with EBV genes. Figure 3 shows the gene interaction network between EBV genes and human hub genes.

### Important human gene pathways

Apoptosis, Lysosome, Jak-STAT signaling pathway, and Phosphatidylinositol signaling system are significant human gene pathways. Those four pathways achieved significant correlation with BALF4 and/or BALF5 in both Pearson correlation analysis and sCCA.

In particular, BALF4 achieved positive correlation with the majority of the individual genes in Jak-STAT signaling pathway (Supplementary Table 9) and Phosphatidylinositol signaling system (Supplementary Table 10).

## DISCUSSION

In this study, we used a series of biostatistics and bioinformatics tools and methods (e.g., DESeq2 gene differential expression analysis, MEGENA gene co-expression analysis, GAGE pathway analysis, principle component analysis, Pearson correlation analysis, and sCCA analysis) to reveal the relationship of EBV and human GC gene expression. The aim of our study is to dissect this relationship at the single EBV gene level. This task was challenging as we tried to correlate two transcriptomes, one from EBV and one from human, and hence the potential data space (number of potentially correlative pairs) runs hundreds of thousands of dimensions. Therefore, we used several tools to try to reduce the dimensionality so that the problem of multiple testing can be alleviated. For example, we used MEGENA gene co-expression analysis and PCA, to extract the most essential information from genome-wide gene expression data.

**Figure 4:**
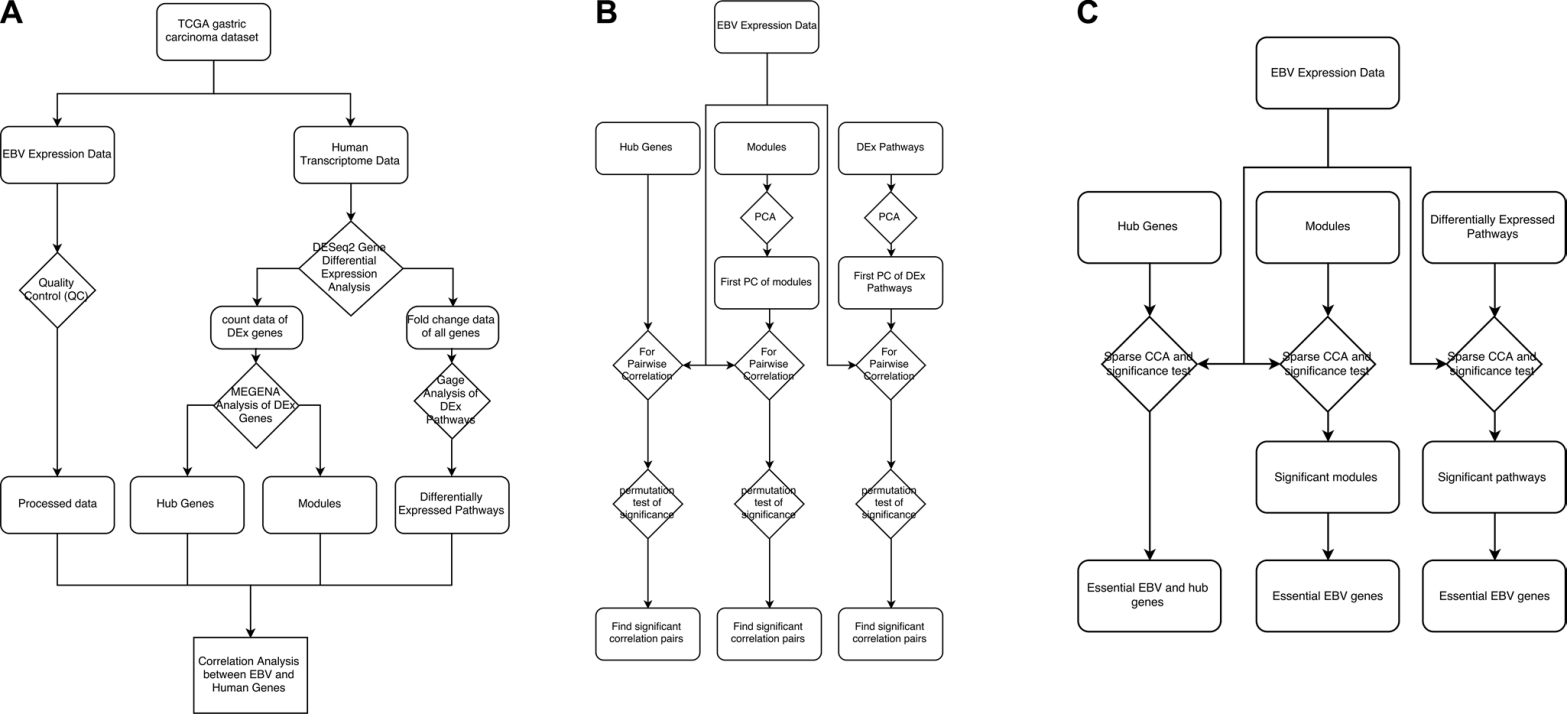
Data analysis workflow

We then applied both Pearson correlation and sCCA to find the relationship of EBV gene expression with human GC gene modules, hub genes and pathways. For correlation analysis, sCCA was more sensitive than Pearson correlation analysis and identified more significant results. However, the results from Pearson correlation analysis are easier to interpret as it was performed pairwise so that the intensity of correlation between an EBV gene and a human gene can be conveniently measured by the correlation coefficient and the statistical significance by the *p* value. Overall, most human gene modules and pathways as identified/extracted from our first phase differential expression analysis (Figure 4A) were found to be significantly correlated with EBV genes at the significance level of FDR < 0.1, which, as expected, suggested that EBV genes did have a strong relationship with human GC cell gene expression.

More importantly, LMP-1, BALF2 and BALF1 are the most significant EBV genes correlating with human gene modules and hub genes. LMP-1 encodes the latent membrane protein-1, which is an oncoprotein and can lead to deregulation of cell growth. Several studies showed that its expression was related with many cancers such as nasopharyngeal carcinoma and Hodgkin's Lymphoma [21–24]. BALF1 encodes anti-apoptotic cellular Bcl2 homologs that can suppress cell apoptosis. Its expression was also found to be related with cancers such as nasopharyngeal carcinoma [25, 26]. For BALF2, its relationship with cancers was not reported before and our study is the first one to show its potential association with gastric carcinoma.

At the human gene side, the hub gene CNTD2 was correlated with the EBV gene BALF1. CNTD2 encodes cyclin N-terminal domain containing 2 and was shown to be associated with methylation by nicotine exposure [27]. It was also involved in 84 cancer networks (The Cancer Network Galaxy/TCNG, http://tcng.hgc.jp/). Another human hub gene, VANGL2, which was correlated with the EBV gene LMP-1, encodes a membrane protein involved in the regulation of planar cell polarity and its expression was shown to be related to breast cancer [28–30].

Two EBV genes, BALF4 and BALF5, achieved significant correlation with four human gene pathways, i.e., Lysosome, Jak-STAT signaling pathway, Phosphatidylinositol signaling system, and Apoptosis. Studies showed that BALF4 and BALF5 were essential to EBV's replication and infection to human cells. BALF4 encodes a protein, gp110, which can dramatically enhance the ability of EBV to infect human cells [31]. BALF5 encodes the DNA polymerase that is essential to the virus' replication [31].

Lysosome regulates cell death and is related to cell canceration [32]. Apoptosis is a very important process and needs to be regulated precisely. Both up- and down regulation of this pathway may contribute to carcinogenesis [33]. Another pathway, Jak-STAT signaling pathway, has been shown to be essential in gastric carcinoma and other cancers [34, 35]. Interestingly, we found a positive correlation between the BALF4 gene and the majority of individual genes in the Jak-STAT signaling pathway (Supplementary Table 9), which suggests that the EBV gene BALF4 alone may play an important role in the upregulation of this pathway in EBV+ vs. EBV− subjects.

The pathway Phosphatidylinositol signaling system is an intricate network of enzymes and phospholipid messengers, and is a crucial regulator of most cellular processes. Upregulation of this pathway may lead to cancers [36, 37]. Interestingly, upregulation of this pathway may be caused by the mutation of PIK3CA (the alpha-isoform of the regulatory subunit of PI3K) [37], which is an important molecular character of EBV+ human GC [4]. In our study, the BALF4 gene also achieved a positive correlation with most of individual genes in this pathway (Supplementary Table 10). Therefore, BALF4 may be the key responsible EBV gene for upregulation of this pathway in EBV+ vs. EBV− samples in our study.

In summary, we performed a study to analyze the interaction between EBV genes and human GC gene expression. Our results indicated that several EBV genes, LMP-1, BALF1, BALF2, BALF4 and BALF5, may interact with the expression of human GC genes, e.g., the CNTD2 and VANGL2, and the gastric cancer-related pathways, e.g., Jak-STAT signaling and phosphatidylinositol signaling system. Our study, by determining the above key interactions, provided new mechanistic insights into the EBV−related GC from the perspective of cross-talk between individual EBV genes and the specific human genes/pathways. Treatment schemes, e.g., new vaccines, medicines and screening kits, targeted at these important EBV genes' products and their interactive partners at the human gene side may be developed to more effectively prevent EBV−related gastric cancer.

## MATERIALS AND METHODS

### Overall workflow

The overall workflow of the study is shown in Figure 4. The workflow is broken down into three parts, feature extraction (Figure 4A, the left panel of the figure), univariate correlation analysis (Figure 4B, the middle panel of the figure) and multivariate correlation analysis (Figure 4C, the right panel of the figure). In feature extraction (Figure 4A), we quantified EBV gene expression and identified those human genes/pathways differentially expressed in EBV+ vs. EBV− samples. Using the expression data of the DEGs, we also extracted the hub genes and gene modules through MEGENA (Multiscale Embedded Gene Co-expression Network Analysis) analysis.

As shown in Figure 4B, we then correlated EBV gene expression data with the human GC expression features extracted in the feature selection step. EBV gene expression was correlated with the hub genes directly. For correlating with the human gene modules and human DEx pathways, we first used PCA (Principle Component Analysis) to extract the first PC of each module and the first PC of each DEx pathway and then correlate the EBV genes with the first PC (Figure 4B). The PCA analysis served to reduce the dimensionality of the human gene expression data and alleviate the problem of multiple testing.

The above correlation was further performed using multivariate correlation approach, i.e., the sparse canonical correlation (sCCA) (Figure 4C), where the whole expression matrix of hub genes, gene modules and DEx pathways were correlated with EBV gene expression matrix directly. Through this analysis, we also selected essential EBV genes that explained the major fraction of the canonical correlation (Figure 4C).

We extracted hub genes and modules to capture the key features that explain the major variation of the genes under EBV regulation. A module is a group of genes who have similar behaviors (or strong cross-gene correlations). A hub gene is a gene that has high connections (strong overall correlations) with other genes. Through modules and hub genes, we essentially took advantage of the inter-correlation information between genes and used that information to effectively reduce the dimensionality of our data.

### Gene expression count generation

Raw fastq data for 285 GC (including 260 EBV− GC and 25 EBV+ GC) samples was downloaded from TCGA (The Cancer Genome Atlas). The downloaded samples' sample IDs are shown in Supplementary Table 1.

The raw fastq data were adaptor-trimmed and mapped to hg19 human reference genome using the TopHat Alignment Tool [38] to generate BAM files. We then used a number of Bioconductor packages to process the BAM files into gene count matrix following the procedures listed under http://www.bioconductor.org/help/workflows/rnaseqGene/

EBV transcript quantification was generated following the pipeline as described in Strong et al. [39]. Briefly, alignment was carried out using Novoalign [−o SAM, paired-end, default options] against the EBV B95-8 genome, followed by EBV transcript quantification using the software SAMMate [40].

### Sample selection

In our dataset, we have 25 EBV+ GC samples and 260 EBV− GC samples. To alleviate confounding effects during the process of comparing EBV+ and EBV− samples, we selected EBV− GC samples that are most similar to the 25 EBV+ GC samples in clinical variables.

We first constructed a design matrix based on the clinical variables, which included gender, age at initial pathological diagnosis, race, anatomic neoplasm subdivision (e.g., fundus/body, antrum/distal, cardia/proximal, etc.), histologic diagnosis (e.g., stomach adenocarcinoma diffuse type, stomach adenocarcinoma tubular type, stomach adenocarcinoma not otherwise specified, etc.), adjacent PT staging, adjacent PN staging, adjacent PM staging, adjacent tumor stage, tumor grade, history of other malignancy, presence of Barrett's esophagus, and family history of gastric carcinoma. The design matrix was built with 285 samples arranged in rows and the above variables arranged in columns. Except for age at diagnosis, all the variables were coded as categorical data (i.e., 0, 1, 2, etc.). Missing values were coded as NA.

We then used the R function, “dist()”, to develop a distance matrix using the design matrix. The distance between two samples (x, y) was calculated based on Euclidean distance, $d(x,y)=\sqrt{\sum_{i=1}^{n}{\left(x_{i}-y_{i}\right)}^{2}}$, where *x_i_* and *y_i_* are the values for the *ith* clinical parameter for the two samples.

For each of the 25 EBV+ samples, we selected an EBV− sample that has the shortest distance from the former. As some of the EBV− samples so selected (that are paired with an EBV+ sample) are the same, the total number of selected EBV− samples is 20.

### Data cleaning and normalization

Those EBV genes (transcripts identified and quantified using EBV B95-8 genome as mentioned above) that have 0-count in more than 5 samples were removed from further analysis.

We applied a library size based method for data normalization, as recommended by a Bioconductor package, GAGE (Generally Applicable Gene-set Enrichment for Pathway Analysis) [41]. The library size of each sample is first calculated by adding all the read counts of genes in that sample using R code *libsizes=colSums(Expr)*, where the dataset Expr is the original count matrix with columns corresponding to samples and rows representing genes. Then we calculated the size factor using this formula: ${\text{sf}}_{i}={\text{libsize}}_{i}/\text{exp}\left(\frac{\sum {\text{log}}_{10}{\text{libsize}}_{i}}{n}\right)$, where *sf_i_* is the size factor of the ith sample, *libsize_i_* is the library size of the *ith* sample, and *n* is the number of samples. It can be done using the following R code: *size.factor=libsizes/exp(mean(log(libsizes)))*. Then we divided all gene counts by the size factor of the corresponding sample to make sure that the library size are comparable among each sample, and then added 8 to each read count to prevent 0 counts and make sure all the *log*_2_ transformed read counts will be greater or equal to 3. Then we applied *log*_2_ transformation on all the read counts. The process was done in R using these codes: *expr.norm=t(t(DExExpr)/size.factor)*; *expr.norm=log2(expr.norm+8)*. The normalized counts are used for constructing gene co-expression network and correlation analysis.

### Differential expression (DEx) analysis

We use “DEseq2” package [42] in Bioconductor to identify differentially expressed genes (DEx genes) between EBV+ and EBV− samples. Genes with a raw *p* value less than 1E-6 (so that the Bonferroni corrected *p* value is less than 0.05) were identified as DEx genes.

### Pathway analysis

We used “GAGE” (Generally Applicable Gene-set Enrichment for Pathway Analysis) [41] in Bioconductor to identify DEx pathways in EBV+ vs. EBV− groups. The log_2_ fold change for all genes from the DEx analysis was submitted to GAGE for the analysis. GAGE then calculated the mean and variance of the fold change (in EBV+ vs. EBV− groups) for gene sets (i.e., the pathways) and for the background (i.e., the whole gene list) using each gene's fold change data. The *t* test was performed to compare the log_2_ fold change of a gene set and the background. The *t* test statistics for all pairwise comparisons between a test group sample and a control group sample were summarized into a new statistic. The new statistic follows a gamma distribution, based on which the overall difference of the gene set between the case samples and the control samples was evaluated for statistical significance. Since the information of up and down regulation could also be obtained from the fold-change data, GAGE could also identify a pathway to be up or down regulated [41]. Those pathways that have a *p* value less than 0.05 after Bonferroni correction were treated as significant pathways for further analysis.

### Gene co-expression network analysis of DEx genes

We used R package MEGENA to construct gene co-expression network of the identified DEx genes [15]. The complete workflow of MEGENA contains 4 major steps: Fast Planar Filtered Network construction (FPFNC), Multiscale Clustering Analysis (MCA), Multiscale Hub Analysis (MHA), and Cluster-Trait Association Analysis (CTA) [15]. We used the first three steps to find the clusters and hub genes in human DEx genes.

### PCA (principal component analysis) of gene modules and DEx pathways

We used PCA for dimension reduction of the identified modules and DEx pathways. PCA performs dimension reduction of correlated variables by projecting those variables to several principal components without losing their variabilities. The first principal component (PC) can explain the largest proportion of variability and the second, third and other subsequent components can explain remaining smaller proportions of variability. One can then use the first PC that contains most of the variability to represent the whole dataset without losing much information.

We applied PCA to the expression data of the individual modules constructed by MEGENA and the expression data of the DEx pathways. We used R function “prcomp()” to implement PCA. The input datasets of “prcomp()” are the normalized count data of modules and pathways. We used the first PC extracted from the data for further downstream analyses.

### Pearson correlation analysis

We performed pairwise Pearson correlation analysis 1) between first PC of modules and EBV gene expression data, 2) between first PC of significant pathways and EBV gene expression data, and 3) between hub genes and EBV gene expression data. We performed permutation test (by permuting the sample labels) on the obtained correlation coefficients to infer their statistical significance. We did not adopt the nominal *p* value for the Pearson correlation as it assumes bivariate normal distribution of the data, which may not be true for the RNA-seq data. Those correlative pairs that achieved FDR values < 0.1 were selected for further comparison with the results from sparse canonical correlation analysis. The permutation tests were performed using C++.

### Sparse canonical correlation analysis (sCCA)

We used sCCA to assess the correlation between EBV gene expression data and expression data of a module, between EBV gene expression and expression data of hub genes, and between EBV gene expression data and expression data of significant pathways.

CCA (canonical correlation analysis) is a classical method to obtain correlation of two data matrices. However, classical CCA can only handle the case where sample size *m*_1_ and *m*_2_ is greater than the number of variables *m*_1_ and *m*_2_ in both matrices. In our expression data the sample size *n* is normally less than the number of variables (the genes). Therefore we use sparse CCA instead. Sparse CCA, by adding a penalty on canonical variants before CCA calculation, can handle the situation, when *m*_1_ and *m*_2_ >> *n*.

We used the R package PMA (Penalized Multivariate Analysis) [43] to perform sparse CCA on our datasets and perform permutation test on the calculated canonical correlation coefficients to test their significance. Those that achieved a *p* value of less than 0.1 after FDR correction were treated as significant. We used the function “CCA.permute()” in PMA to select the tuning parameters of sparse CCA and then used the function “CCA()” in PMA to perform sCCA using the parameters selected. “CCA.permute()” and “CCA()” require the input of two matrices to be correlated, with samples in rows and genes in columns. We submitted the normalized count matrices of EBV genes and human gene modules, EBV genes and human hub genes, EBV genes and human gene pathways to the two functions separately for sCCA analysis.

The canonical coefficient of each element feature (e.g., an EBV gene) can be obtained from the result of “CCA()”, which can measure the contribution of an element feature (e.g., a specific EBV gene) to the overall canonical correlation. For example, a feature with a non-zero canonical coefficient means that it contributes substantially to the overall correlation between two matrices. Thus, we selected the genes with non-zero canonical coefficients as “essential” genes.

To make the selection of essential genes robust, we adopted a method from a study by Lin et al [44]. We randomly sampled 23 out of all the 25 EBV+ samples for 100 times. It is expected that if a gene has a significant contribution to the overall canonical correlation, it would achieve a non-zero canonical coefficient in most of samplings. This means that the contribution of one gene to the overall canonical correlation can be quantified by the frequency of times of achieving a non-zero canonical coefficient in the 100 samplings. So the contribution of one gene to the overall canonical correlation can be calculated using this formula [44].

$$g_{i}=\frac{1}{N}\overset{N}{\underset{n=1}{\sum }}I(u_{i,n}\neq 0)$$

where *g_i_* is the importance of gene i, *u_i,n_* is the canonical coefficient of gene i in *n*th sampling, *N* is the total number of sampling, and *I* (*u_i, n_*≠ 0) is an indicator function, which is equal to 1 if *u_i, n_* ≠ 0, otherwise 0. We set a stringent threshold that a gene with *g_i_* > 0.9 will be selected as the essential gene (as indicated in Figure 4C).

### Comparison of analytical results between univariate pearson correlation analysis and sCCA

We compared univariate Pearson correlation analysis and sCCA analysis results. Those that achieved significant results in both analyses were summarized or annotated using DAVID (Database for Annotation, Visualization and Integrated Discovery) [16, 17], which can annotate the genes at the levels of Gene Ontology (GO), KEGG (Kyoto Encyclopedia of Genes and Genomes), SP-PIR (protein information resource) and other functional terms. We also drew the graphs to show the interactions between significant EBV genes and human module/hub/pathway genes by their cross-correlation. For that purpose, we used the R function network() in R package MixOmics [45]. The function network() requires a correlation matrix (in our case the Pearson correlation matrix) between two sets of features and can show correlations with different magnitude in different color [45].

## SUPPLEMENTARY TABLES









## References

[1] Bray F, Ren JS, Masuyer E, Ferlay J (2013). Global estimates of cancer prevalence for 27 sites in the adult population in 2008. Int J Cancer.

[2] de Martel C, Forman D, Plummer M (2013). Gastric cancer: epidemiology and risk factors. Gastroenterol Clin North Am.

[3] Correa P (1992). Human gastric carcinogenesis: a multistep and multifactorial process—First American Cancer Society Award Lecture on Cancer Epidemiology and Prevention. Cancer Res.

[4] Cancer Genome Atlas Research Network (2014). Comprehensive molecular characterization of gastric adenocarcinoma. Nature.

[5] Ko YH (2015). EBV and human cancer. Exp Mol Med.

[6] Hu H, Luo ML, Desmedt C, Nabavi S, Yadegarynia S, Hong A, Konstantinopoulos PA, Gabrielson E, Hines-Boykin R, Pihan G, Yuan X, Sotirious C, Dittmer DP (2016). Epstein-Barr Virus Infection of Mammary Epithelial Cells Promotes Malignant Transformation. EBioMedicine.

[7] Schmitz R, Ceribelli M, Pittaluga S, Wright G, Staudt LM (2014). Oncogenic mechanisms in Burkitt lymphoma. Cold Spring Harb Perspect Med.

[8] Zazula M, Ferreira AM, Czopek JP, Kolodziejczyk P, Sinczak-Kuta A, Klimkowska A, Wojcik P, Okon K, Bialas M, Kulig J, Stachura J (2006). CDH1 gene promoter hypermethylation in gastric cancer: relationship to Goseki grading, microsatellite instability status, and EBV invasion. Diagn Mol Pathol.

[9] Vauhkonen M, Vauhkonen H, Sipponen P (2006). Pathology and molecular biology of gastric cancer. Best Pract Res Clin Gastroenterol.

[10] Chang MS, Uozaki H, Chong JM, Ushiku T, Sakuma K, Ishikawa S, Hino R, Barua RR, Iwasaki Y, Arai K, Fujii H, Nagai H, Fukayama M (2006). CpG island methylation status in gastric carcinoma with and without infection of Epstein-Barr virus. Clin Cancer Res.

[11] Lee HS, Chang MS, Yang HK, Lee BL, Kim WH (2004). Epstein-barr virus-positive gastric carcinoma has a distinct protein expression profile in comparison with epstein-barr virus-negative carcinoma. Clin Cancer Res.

[12] Kim J, Lee HS, Bae SI, Lee YM, Kim WH (2005). Silencing and CpG island methylation of GSTP1 is rare in ordinary gastric carcinomas but common in Epstein-Barr virus-associated gastric carcinomas. Anticancer Res.

[13] Qu Y, Dang S, Hou P (2013). Gene methylation in gastric cancer. Clin Chim Acta.

[14] Kusano M, Toyota M, Suzuki H, Akino K, Aoki F, Fujita M, Hosokawa M, Shinomura Y, Imai K, Tokino T (2006). Genetic, epigenetic, and clinicopathologic features of gastric carcinomas with the CpG island methylator phenotype and an association with Epstein-Barr virus. Cancer.

[15] Song WM, Zhang B (2015). Multiscale Embedded Gene Co-expression Network Analysis. PLoS Comput Biol.

[16] Dennis G, Sherman BT, Hosack DA, Yang J, Gao W, Lane HC, Lempicki RA (2003). DAVID: Database for Annotation, Visualization, and Integrated Discovery. Genome Biol.

[17] Huang da W, Sherman BT, Lempicki RA (2009). Systematic and integrative analysis of large gene lists using DAVID bioinformatics resources. Nat Protoc.

[18] Waseem T (2010). Commentary: Ghrelin's role in gastrointestinal tract cancer. Surg Oncol.

[19] Waseem T, Javaid UR, Ahmad F, Azam M, Qureshi MA (2008). Role of ghrelin axis in colorectal cancer: a novel association. Peptides.

[20] Duxbury MS, Waseem T, Ito H, Robinson MK, Zinner MJ, Ashley SW, Whang EE (2003). Ghrelin promotes pancreatic adenocarcinoma cellular proliferation and invasiveness. Biochem Biophys Res Commun.

[21] Tsao SW, Tramoutanis G, Dawson CW, Lo AK, Huang DP (2002). The significance of LMP1 expression in nasopharyngeal carcinoma. Semin Cancer Biol.

[22] Hannigan A, Wilson JB (2010). Evaluation of LMP1 of Epstein-Barr virus as a therapeutic target by its inhibition. Mol Cancer.

[23] Kondo S, Wakisaka N, Muramatsu M, Zen Y, Endo K, Murono S, Sugimoto H, Yamaoka S, Pagano JS, Yoshizaki T (2011). Epstein-Barr virus latent membrane protein 1 induces cancer stem/progenitor-like cells in nasopharyngeal epithelial cell lines. J Virol.

[24] Herling M, Rassidakis GZ, Medeiros LJ, Vassilakopoulos TP, Kliche KO, Nadali G, Viviani S, Bonfante V, Giardini R, Chilosi M, Kittas C, Gianni AM, Bonadonna G (2003). Expression of Epstein-Barr virus latent membrane protein-1 in Hodgkin and Reed-Sternberg cells of classical Hodgkin's lymphoma: associations with presenting features, serum interleukin 10 levels, and clinical outcome. Clin Cancer Res.

[25] Cabras G, Decaussin G, Zeng Y, Djennaoui D, Melouli H, Broully P, Bouguermouh AM, Ooka T (2005). Epstein-Barr virus encoded BALF1 gene is transcribed in Burkitt's lymphoma cell lines and in nasopharyngeal carcinoma’s biopsies. J Clin Virol.

[26] Bellows DS, Howell M, Pearson C, Hazlewood SA, Hardwick JM (2002). Epstein-Barr virus BALF1 is a BCL-2-like antagonist of the herpesvirus antiapoptotic BCL-2 proteins. J Virol.

[27] Chhabra D, Sharma S, Kho AT, Gaedigk R, Vyhlidal CA, Leeder JS, Morrow J, Carey VJ, Weiss ST, Tantisira KG, Demeo DL (2014). Fetal lung and placental methylation is associated with in utero nicotine exposure. Epigenetics.

[28] Hatakeyama J, Wald JH, Printsev I, Ho HY, Carraway KL (2014). Vangl1 and Vangl2: planar cell polarity components with a developing role in cancer. Endocr Relat Cancer.

[29] Puvirajesinghe TM, Bertucci F, Jain A, Scerbo P, Belotti E, Audebert S, Sebbagh M, Lopez M, Brech A, Finetti P, Charafe-Jauffret E, Chaffanet M, Castellano R (2016). Identification of p62/SQSTM1 as a component of non-canonical Wnt VANGL2-JNK signalling in breast cancer. Nat Commun.

[30] Belotti E, Puvirajesinghe TM, Audebert S, Baudelet E, Camoin L, Pierres M, Lasvaux L, Ferracci G, Montcouquiol M, Borg JP (2012). Molecular characterisation of endogenous Vangl2/Vangl1 heteromeric protein complexes. PLoS ONE.

[31] Neuhierl B, Feederle R, Hammerschmidt W, Delecluse HJ (2002). Glycoprotein gp110 of Epstein-Barr virus determines viral tropism and efficiency of infection. Proc Natl Acad Sci USA.

[32] Kroemer G, Jaattela M (2005). Lysosomes and autophagy in cell death control. Nat Rev Cancer.

[33] Lowe SW, Lin AW (2000). Apoptosis in cancer. Carcinogenesis.

[34] Thomas SJ, Snowden JA, Zeidler MP, Danson SJ (2015). The role of JAK/STAT signalling in the pathogenesis, prognosis and treatment of solid tumours. Br J Cancer.

[35] Yeh CM, Chang LY, Lin SH, Chou JL, Hsieh HY, Zeng LH, Chuang SY, Wang HW, Dittner C, Lin CY, Lin JM, Huang YT, Ng EK (2016). Epigenetic silencing of the NR4A3 tumor suppressor, by aberrant JAK/STAT signaling, predicts prognosis in gastric cancer. Sci Rep.

[36] Vivanco I, Sawyers CL (2002). The phosphatidylinositol 3-Kinase AKT pathway in human cancer. Nat Rev Cancer.

[37] Bunney TD, Katan M (2010). Phosphoinositide signalling in cancer: beyond PI3K and PTEN. Nat Rev Cancer.

[38] Trapnell C, Roberts A, Goff L, Pertea G, Kim D, Kelley DR, Pimentel H, Salzberg SL, Rinn JL, Pachter L (2012). Differential gene and transcript expression analysis of RNA-seq experiments with TopHat and Cufflinks. Nat Protoc.

[39] Strong MJ, Xu G, Coco J, Baribault C, Vinay DS, Lacey MR, Strong AL, Lehman TA, Seddon MB, Lin Z, Concha M, Baddoo M, Ferris M (2013). Differences in gastric carcinoma microenvironment stratify according to EBV infection intensity: implications for possible immune adjuvant therapy. PLoS Pathog.

[40] Xu G, Deng N, Zhao Z, Judeh T, Flemington E, Zhu D (2011). SAMMate: a GUI tool for processing short read alignments in SAM/BAM format. Source Code Biol Med.

[41] Luo W, Friedman MS, Shedden K, Hankenson KD, Woolf PJ (2009). GAGE: generally applicable gene set enrichment for pathway analysis. BMC Bioinformatics.

[42] Love MI, Huber W, Anders S (2014). Moderated estimation of fold change and dispersion for RNA-seq data with DESeq2. Genome Biol.

[43] Witten DM, Tibshirani R, Hastie T (2009). A penalized matrix decomposition, with applications to sparse principal components and canonical correlation analysis. Biostatistics.

[44] Lin D, Calhoun VD, Wang YP (2014). Correspondence between fMRI and SNP data by group sparse canonical correlation analysis. Med Image Anal.

[45] Cao KL, Rohart F, Gonzalez I, Dejean S, Gautier B, Bartolo F (2016). mixOmics: Omics Data Integration Project. R package version. 6 1 1.

