# Supplementary material for: Interaction of Epstein-Barr virus genes with human gastric carcinoma transcriptome
Source: Oncotarget. 2017 Mar 21;8(24):38399–412. doi: 10.18632/oncotarget.16417 (PMC5503541; doi:10.18632/oncotarget.16417)
Supplement: Supplementary file 1 [file oncotarget-08-38399-s001.pdf]

Interaction of Epstein-Barr virus genes with human gastric carcinoma transcriptome

Supplementary Materials

Supplementary Table 1: TCGA sample IDs of downloaded samples. See Supplementary_Table_1

Supplementary Table 2: Symbols of differentially expressed genes. See Supplementary_Table_2

Supplementary Table 3: Modules extracted from the DEx human genes

Module number	Number of genes within each module
1	19
2	90
3	12
4	32
5	103
6	139
7	45
8	75
9	65
10	16
11	38
12	54
13	50
14	54
15	20
16	12
17	24
18	39
19	14
20	57
21	24
22	25
23	29
24	10
25	15
26	17
27	12

Supplementary Table 4: Hub genes identified from the DEx human Genes

AC092669.3	DENND2C	KLRK1	RP11-30J20.1	TMEM55A
AC133785.1	DLX6-AS1	LARP6	RP11-323F24.1	TTLL7
AC138430.4	DUOX1	MAPK8IP1	RP11-347C12.3	TUBB6
AGAP11	FASLG	MIB2	RP11-385J1.2	ULK2
AXDND1	FBXO27	MXRA7	RP11-529A4.4	UPK1B
C19orf54	GALNT15	NDNF	RP11-776H12.1	VANGL2
C1orf115	GBP4	NKX2-5	RP11-78F17.1	WARS
CAV2	GREB1	OCA2	SCML2P1	WASF3
CCR5	GZMH	PLA2R1	SCN3B	XPNPEP2
CD38	HOXA11	PRF1	SLAMF7	ZNF317
CHADL	HOXA11-AS	PRKCDBP	SLC16A14	ZNF470
CHP2	HOXC10	PRPH	SLC22A3	
CISD1	HOXD-AS1	PYGO1	SLC25A21	
CLCN1	IDO1	RAB34	SLC30A10	
CNTD2	IGF2BP1	RASSF8	SPATA13	
CSAG2	KCNB2	RIPPLY2	SRPX	
CTB-25B13.12	KCNJ12	RNF144A	SUGP2	
CTD-2105E13.6	KCNK15	RP11-108M12.3	SYT1	
CXCL10	KIAA1377	RP11-14N7.2	TCF7L1	
CXCL11	KIF26A	RP11-215A19.1	TMEM220	

Supplementary Table 5: The percent of variation explained by the PC1 of each module

Modules	Percent of variation explained by PC1
1	51.5%
2	54.3%
3	62.8%
4	58.9%
5	52.7%
6	44.5%
7	50.7%
8	51.7%
9	50.1%
10	55.8%
11	50.3%
12	47.9%
13	44.7%
14	46.8%
15	56.6%
16	61.8%
17	46.5%
18	60.1%
19	74.5%
20	53.7%
21	66.6%
22	53.0%
23	46.8%
24	64.9%
25	66.3%
26	50.4%
27	55.4%

Supplementary Table 6: The percent of variation explained by the PC1 of each pathway

Pathways	Percent of variation explained by PC1
1	36.3%
2	36.3%
3	28.0%
4	29.4%
5	28.3%
6	32.8%
7	28.7%
8	40.0%
9	36.2%
10	63.4%
11	30.7%
12	30.6%
13	23.0%
14	29.2%
15	33.6%
16	38.5%
17	22.1%
18	35.2%
19	35.6%
20	29.9%
21	26.3%
22	48.5%
23	26.1%
24	29.6%
25	40.7%
26	27.2%
27	18.4%
28	31.6%
29	28.2%

Supplementary Table 7: DAVID annotation of human gene module 5

Category	Functional terms	FDR
UP_KEYWORDS	Membrane	4.00E-04
UP_KEYWORDS	Transmembrane	0.001
UP_KEYWORDS	Transmembrane helix	0.002
UP_SEQ_FEATURE	transmembrane region	0.007
UP_SEQ_FEATURE	topological domain:Cytoplasmic	0.020
UP_SEQ_FEATURE	glycosylation site:N-linked (GlcNAc...)	0.027
UP_KEYWORDS	Glycoprotein	0.032
UP_KEYWORDS	Cell membrane	0.046
GOTERM_CC_DIRECT	GO:0045202~synapse	0.047
UP_KEYWORDS	Transport	0.054
UP_KEYWORDS	Potassium channel	0.059
GOTERM_CC_DIRECT	GO:0005886~plasma membrane	0.059
UP_KEYWORDS	Voltage-gated channel	0.062
UP_KEYWORDS	Ion transport	0.062
UP_KEYWORDS	Ion channel	0.069
GOTERM_CC_DIRECT	GO:0016021~integral component of membrane	0.073

Supplementary Table 8: DAVID annotation of human gene module 12

Category	Term	FDR
SP_PIR_KEYWORDS	calcium	0.008
SP_PIR_KEYWORDS	signal	0.049
SP_PIR_KEYWORDS	Secreted	0.061
INTERPRO	IPR011992:EF-Hand type	0.068
GOTERM_MF_FAT	GO:0005509~calcium ion binding	0.071
INTERPRO	IPR018247:EF-HAND 1	0.075
INTERPRO	IPR018248:EF hand	0.086
BIOCARTA	Ghrelin Pathway: Ghrelin	0.087
SP_PIR_KEYWORDS	glycoprotein	0.087
SP_PIR_KEYWORDS	cleavage on pair of basic residues	0.097

Supplementary Table 9: Correlation between BALF4 and individual genes in JAK-STAT Signaling Pathway

Gene	Correlation coefficients	FDR
CREBBP	0.747	0.003
GRB2	0.862	0.003
MCL1	0.59	0.003
JAK1	0.63	0.02
MTOR	0.592	0.027
STAM	0.076	0.044
AOX1	0.444	0.067
STAT1	0.465	0.067
AKT1	0.449	0.077
BCL2L1	0.458	0.077
CDKN1A	0.473	0.077
HRAS	−0.428	0.081
SOCS1	0.26	0.09
BCL2	0.391	0.096

Supplementary Table 10: Correlation between BALF4 and individual genes in phosphatidylinositol signaling system

Genes	Correlation coefficients	FDR
PI4KA	0.764	< 0.001
PIP5K1A	0.691	< 0.001
INPP5D	0.673	0.002
IPPK	0.686	0.002
INPP4A	0.687	0.003
MTM1	0.589	0.018
PIKFYVE	0.508	0.018
INPP5B	0.538	0.022
IP6K1	0.563	0.022
ITPR1	0.494	0.03
PPIP5K1	0.502	0.037
PRKCA	0.445	0.062
DGKA	0.423	0.093
ITPK1	0.396	0.093
PLCB2	0.41	0.093

Supplementary Table 11: Selected EBV+ and EBV− samples. See Supplementary_Table_11
