# Supplementary material for: Interaction of Epstein-Barr virus genes with human gastric carcinoma transcriptome
Source: Oncotarget. 2017 Mar 21;8(24):38399–412. doi: 10.18632/oncotarget.16417 (PMC5503541; doi:10.18632/oncotarget.16417)
Supplement: Supplementary file 2 [file oncotarget-08-38399-s002.docx]

**Supplemental Table 1: TCGA sample IDs of downloaded samples**

| TCGA-B7-5816 | TCGA-BR-6802 | TCGA-BR-8371 | TCGA-BR-A4J7 | TCGA-CG-4436 | TCGA-D7-6526 | TCGA-HF-7134 |
| --- | --- | --- | --- | --- | --- | --- |
| TCGA-B7-5818 | TCGA-BR-6803 | TCGA-BR-8372 | TCGA-BR-A4J8 | TCGA-CG-4437 | TCGA-D7-6527 | TCGA-HF-7136 |
| TCGA-BR-4187 | TCGA-BR-6852 | TCGA-BR-8373 | TCGA-BR-A4J9 | TCGA-CG-4438 | TCGA-D7-6528 | TCGA-HJ-7597 |
| TCGA-BR-4191 | TCGA-BR-7196 | TCGA-BR-8380 | TCGA-BR-A4PD | TCGA-CG-4440 | TCGA-D7-6815 | TCGA-HU-8238 |
| TCGA-BR-4201 | TCGA-BR-7197 | TCGA-BR-8381 | TCGA-BR-A4PE | TCGA-CG-4441 | TCGA-D7-6817 | TCGA-HU-8243 |
| TCGA-BR-4253 | TCGA-BR-7703 | TCGA-BR-8382 | TCGA-BR-A4PF | TCGA-CG-4442 | TCGA-D7-6818 | TCGA-HU-8244 |
| TCGA-BR-4255 | TCGA-BR-7704 | TCGA-BR-8384 | TCGA-BR-A4QI | TCGA-CG-4443 | TCGA-D7-6820 | TCGA-HU-8602 |
| TCGA-BR-4256 | TCGA-BR-7707 | TCGA-BR-8483 | TCGA-BR-A4QL | TCGA-CG-4444 | TCGA-D7-6822 | TCGA-HU-8604 |
| TCGA-BR-4257 | TCGA-BR-7715 | TCGA-BR-8484 | TCGA-BR-A4QM | TCGA-CG-4449 | TCGA-D7-8570 | TCGA-HU-8608 |
| TCGA-BR-4267 | TCGA-BR-7716 | TCGA-BR-8485 | TCGA-CD-5798 | TCGA-CG-4460 | TCGA-D7-8572 | TCGA-HU-8610 |
| TCGA-BR-4279 | TCGA-BR-7717 | TCGA-BR-8486 | TCGA-CD-5799 | TCGA-CG-4462 | TCGA-D7-8573 | TCGA-HU-A4G2 |
| TCGA-BR-4280 | TCGA-BR-7722 | TCGA-BR-8487 | TCGA-CD-5800 | TCGA-CG-4465 | TCGA-D7-8574 | TCGA-HU-A4G3 |
| TCGA-BR-4292 | TCGA-BR-7723 | TCGA-BR-8588 | TCGA-CD-5801 | TCGA-CG-4466 | TCGA-D7-8575 | TCGA-HU-A4G6 |
| TCGA-BR-4294 | TCGA-BR-7851 | TCGA-BR-8589 | TCGA-CD-5803 | TCGA-CG-4469 | TCGA-D7-8576 | TCGA-HU-A4G8 |
| TCGA-BR-4357 | TCGA-BR-7901 | TCGA-BR-8590 | TCGA-CD-5804 | TCGA-CG-4472 | TCGA-D7-8578 | TCGA-HU-A4G9 |
| TCGA-BR-4361 | TCGA-BR-7957 | TCGA-BR-8591 | TCGA-CD-5813 | TCGA-CG-4474 | TCGA-D7-8579 | TCGA-HU-A4GC |
| TCGA-BR-4362 | TCGA-BR-7958 | TCGA-BR-8592 | TCGA-CD-8524 | TCGA-CG-4475 | TCGA-D7-A4YT | TCGA-HU-A4GF |
| TCGA-BR-4363 | TCGA-BR-7959 | TCGA-BR-8676 | TCGA-CD-8525 | TCGA-CG-4476 | TCGA-D7-A4YU | TCGA-HU-A4GH |
| TCGA-BR-4366 | TCGA-BR-8058 | TCGA-BR-8677 | TCGA-CD-8526 | TCGA-CG-4477 | TCGA-D7-A4YV | TCGA-HU-A4GJ |
| TCGA-BR-4367 | TCGA-BR-8059 | TCGA-BR-8678 | TCGA-CD-8527 | TCGA-CG-5716 | TCGA-D7-A4YX | TCGA-HU-A4GN |
| TCGA-BR-4368 | TCGA-BR-8060 | TCGA-BR-8679 | TCGA-CD-8528 | TCGA-CG-5717 | TCGA-D7-A4YY | TCGA-HU-A4GP |
| TCGA-BR-4369 | TCGA-BR-8077 | TCGA-BR-8680 | TCGA-CD-8529 | TCGA-CG-5718 | TCGA-D7-A4Z0 | TCGA-HU-A4GT |
| TCGA-BR-4370 | TCGA-BR-8078 | TCGA-BR-8682 | TCGA-CD-8530 | TCGA-CG-5719 | TCGA-EQ-8122 | TCGA-HU-A4GU |
| TCGA-BR-4371 | TCGA-BR-8080 | TCGA-BR-8683 | TCGA-CD-8531 | TCGA-CG-5720 | TCGA-EQ-A4SO | TCGA-HU-A4GX |
| TCGA-BR-6452 | TCGA-BR-8081 | TCGA-BR-8686 | TCGA-CD-8532 | TCGA-CG-5721 | TCGA-F1-6177 | TCGA-HU-A4GY |
| TCGA-BR-6453 | TCGA-BR-8284 | TCGA-BR-8687 | TCGA-CD-8533 | TCGA-CG-5722 | TCGA-F1-6874 | TCGA-HU-A4H0 |
| TCGA-BR-6454 | TCGA-BR-8286 | TCGA-BR-8690 | TCGA-CD-8534 | TCGA-CG-5723 | TCGA-F1-6875 | TCGA-HU-A4H2 |
| TCGA-BR-6455 | TCGA-BR-8289 | TCGA-BR-A44T | TCGA-CD-8535 | TCGA-CG-5724 | TCGA-F1-A448 | TCGA-HU-A4H3 |
| TCGA-BR-6456 | TCGA-BR-8291 | TCGA-BR-A452 | TCGA-CD-8536 | TCGA-CG-5725 | TCGA-FP-7735 | TCGA-HU-A4H4 |
| TCGA-BR-6457 | TCGA-BR-8295 | TCGA-BR-A453 | TCGA-CD-A486 | TCGA-CG-5726 | TCGA-FP-7829 | TCGA-HU-A4H5 |
| TCGA-BR-6458 | TCGA-BR-8296 | TCGA-BR-A4CR | TCGA-CD-A487 | TCGA-CG-5732 | TCGA-FP-7916 | TCGA-HU-A4H6 |
| TCGA-BR-6563 | TCGA-BR-8297 | TCGA-BR-A4CS | TCGA-CD-A489 | TCGA-CG-5734 | TCGA-FP-7998 | TCGA-HU-A4H8 |
| TCGA-BR-6564 | TCGA-BR-8361 | TCGA-BR-A4IU | TCGA-CD-A48C | TCGA-D7-5577 | TCGA-FP-8099 | TCGA-HU-A4HB |
| TCGA-BR-6565 | TCGA-BR-8362 | TCGA-BR-A4IV | TCGA-CD-A4MG | TCGA-D7-5578 | TCGA-FP-8209 | TCGA-HU-A4HD |
| TCGA-BR-6566 | TCGA-BR-8363 | TCGA-BR-A4IY | TCGA-CD-A4MH | TCGA-D7-6518 | TCGA-FP-8210 | TCGA-IN-7806 |
| TCGA-BR-6705 | TCGA-BR-8364 | TCGA-BR-A4IZ | TCGA-CD-A4MI | TCGA-D7-6519 | TCGA-FP-8211 | TCGA-IN-7808 |
| TCGA-BR-6706 | TCGA-BR-8365 | TCGA-BR-A4J1 | TCGA-CD-A4MJ | TCGA-D7-6520 | TCGA-FP-8631 | TCGA-IN-8462 |
| TCGA-BR-6707 | TCGA-BR-8366 | TCGA-BR-A4J2 | TCGA-CG-4301 | TCGA-D7-6521 | TCGA-FP-A4BE | TCGA-IN-8663 |
| TCGA-BR-6709 | TCGA-BR-8367 | TCGA-BR-A4J4 | TCGA-CG-4304 | TCGA-D7-6522 | TCGA-HF-7131 | TCGA-IP-7968 |
| TCGA-BR-6710 | TCGA-BR-8368 | TCGA-BR-A4J5 | TCGA-CG-4305 | TCGA-D7-6524 | TCGA-HF-7132 |  |
| TCGA-BR-6801 | TCGA-BR-8369 | TCGA-BR-A4J6 | TCGA-CG-4306 | TCGA-D7-6525 | TCGA-HF-7133 |  |
