# Supplementary material for: Interaction of Epstein-Barr virus genes with human gastric carcinoma transcriptome
Source: Oncotarget. 2017 Mar 21;8(24):38399–412. doi: 10.18632/oncotarget.16417 (PMC5503541; doi:10.18632/oncotarget.16417)
Supplement: Supplementary file 3 [file oncotarget-08-38399-s003.docx]

**Supplemental Table 2: Symbols of differentially expressed genes**

Upregulated genes (n= 189)

| ARID2 | ANKRD32 | P2RY11 | PAQR6 | FDXR | GPR171 | GBP1 | RP11-834C11.4 | GBP4 | IGHV3-66 |
| --- | --- | --- | --- | --- | --- | --- | --- | --- | --- |
| HNRPLL | RFX5 | AEN | BBC3 | GPX2 | SYNE2 | TCAP | RP11-611O2.5 | WARS | IGHV3-73 |
| ZNF317 | TMEM143 | SPATA13 | RNF213 | PVRL4 | RP11-1094M14.8 | CTD-3137H5.1 | GZMB | AE000661.37 | CAMK2N2 |
| RAB8A | ZNF618 | HIP1R | DPY19L1 | HOXC4 | RP11-1094M14.5 | KIR3DL2 | FASLG | RP11-39K24.7 | NEB |
| TRIM26 | GSAP | APOBEC3F | SAMHD1 | AC008964.1 | MDM2 | MANEAL | RP1-290I10.6 | GBP5 | A4GNT |
| SUGP2 | PKP4 | RCN1 | SESN2 | ATF5 | PGAM1P7 | AC092580.4 | C11orf93 | SLC6A14 | CXCL10 |
| ZNF227 | TRIAP1 | ARHGEF3 | UBE2L6 | PVRL1 | PLCH1 | CXCR6 | AC002331.1 | CXCL11 | IGHV3-13 |
| ATXN7L3B | ZNF222 | RANBP6 | SESN1 | DDB2 | PRKCQ | HS3ST1 | SYCE2 | DAPL1 | IDO1 |
| TRIM5 | RAPH1 | B2M | ITGAE | RCL1 | USP30 | AC006129.4 | CYSLTR2 | SLC28A3 | RTBDN |
| LINS | CASP4 | TAP2 | APOL3 | IL18R1 | FAS | CD8A | AMH | ZNF683 | IFNG |
| ZNF267 | TCF20 | C12orf5 | CTB-25B13.12 | ENC1 | CCL5 | RUFY4 | ALDH3A1 | LEMD1 | SUSD4 |
| PAPD4 | MB21D1 | TRIM24 | TAP1 | KLRK1 | XCL2 | CCRL1 | MSX2 | ABCA12 | RP11-723O4.9 |
| DNMT1 | IQCB1 | BAX | CD40 | PTPN22 | SLC9A7 | SLAMF7 | CX3CL1 | SOX2 | CXCR2P1 |
| PSPC1 | GCA | ANKRD24 | CD101 | FCGR1B | CIITA | RP11-611O2.3 | JAK2 | KCNJ15 | RP11-291B21.2 |
| C19orf54 | FUT8 | TRIM22 | RP11-876N24.5 | LAP3 | APOBEC3H | GZMA | COL22A1 | CTD-2288O8.1 | KHSRPP1 |
| DUT | CD47 | ATP2C1 | IRF1 | IRAK3 | CD244 | FCRL6 | SUCNR1 | CXCL9 | BPIFB1 |
| SKA2 | LRIG3 | CTSC | ATP2A1 | STAT1 | HAPLN3 | OR2I1P | CD38 | LGALS17A | SLC26A9 |
| MOV10 | CHAF1B | PRPS2 | ETV7 | FHDC1 | RP11-651P23.2 | GZMH | AZGP1 | IGHV2-26 | DLGAP1 |
| TAPBP | BRIP1 | PAQR4 | APOL6 | CCR5 | PRF1 | RP11-638I2.9 | SPTSSB | CD274 |  |

Downregulated genes (n = 750)

| ABCA9 | B3GALNT1 | CKMT2 | EVX1 | HOXA10 | LRRC34 | NXPE2 | RGN | RP11-98G7.1 | TCF7L1 |
| --- | --- | --- | --- | --- | --- | --- | --- | --- | --- |
| ABCG5 | B3GNT8 | CLCN1 | F10 | HOXA11 | LRRC43 | OBSL1 | RHOF | RP4-660H19.1 | TDRD5 |
| ABCG8 | B4GALNT1 | CLDN15 | F12 | HOXA11 | LRRC6 | OCA2 | RIBC2 | RP5-1043L13.1 | TEX15 |
| AC004507.1 | BAIAP3 | CLDN3 | F7 | HOXA2 | LRRC7 | OSBP2 | RIMKLA | RP5-884M6.1 | TEX40 |
| AC007128.1 | BBS5 | CLDN6 | FABP1 | HOXA5 | LRRN1 | OSBPL6 | RIMS4 | RP5-981L23.1 | TF |
| AC007182.6 | BCAS4 | CLEC2L | FABP2 | HOXA9 | LRRN4 | OXGR1 | RIPPLY2 | RPL3P4 | TFAP2C |
| AC008174.3 | BCL2L10 | CLGN | FAH | HOXB | LRRTM1 | OXT | RN7SKP54 | RPRM | TFAP2E |
| AC009236.2 | BDKRB1 | CLPSL2 | FAIM | HOXC | LTK | PAH | RN7SL689P | RSPO4 | TFR2 |
| AC009237.17 | BEND6 | CNDP1 | FAM131C | HOXC10 | LY6K | PANX2 | RND2 | RXFP4 | THNSL2 |
| AC009238.7 | BEST4 | CNTD2 | FAM132A | HOXC12 | M1AP | PAQR5 | RNF126P1 | RYR3 | TIMP4 |
| AC011288.2 | BEX1 | CNTFR | FAM155B | HOXC8 | MAGEA2 | PAX6 | RNF144A | S100A1 | TM4SF20 |
| AC011298.2 | BEX2 | CRABP1 | FAM171A2 | HOXD | MAMSTR | PCDHA11 | RNF144A | SATB2 | TM6SF2 |
| AC011330.12 | BHMT | CRAT | FAM178B | HOXD | MAP10 | PCDHA13 | RNF186 | SBF2 | TMEM117 |
| AC011523.2 | BMP3 | CRHR2 | FAM189A1 | HOXD10 | MAP1LC3A | PCDHAC1 | RP11-108K3.1 | SCIN | TMEM132B |
| AC011897.1 | BMP7 | CRIP3 | FAM211B | HPN | MAP6D1 | PCDHAC2 | RP11-108M12.3 | SCML2P1 | TMEM136 |
| AC013271.3 | BNIP3 | CRLF1 | FAM213A | HRASLS | MAP7D2 | PCDHB2 | RP11-1103G16.1 | SCN2A | TMEM151A |
| AC016735.2 | BNIP3P1 | CSAG1 | FAM222A | HTR1D | MAPK15 | PCDHGA10 | RP11-1134I14.8 | SCN3B | TMEM179 |
| AC018865.8 | BOLA3 | CSAG2 | FAM229B | HTR2C | MAPK8IP1 | PCLO | RP11-138J23.1 | SCN5A | TMEM220 |
| AC018865.9 | BRSK2 | CSAG3 | FAM3B | HYLS1 | MAPRE3 | PCSK1N | RP11-14N7.2 | SCNN1A | TMEM229A |
| AC019100.3 | BTBD11 | CSPG5 | FAM46B | IFNLR1 | MCOLN3 | PDLIM4 | RP11-152P17.2 | SCRN1 | TMEM236 |
| AC026806.2 | C10orf112 | CTA-14H9.5 | FAM5C | IGF2BP1 | MEP1A | PEG10 | RP11-161H23.5 | SEMA3D | TMEM52 |
| AC027612.6 | C11orf70 | CTC-338M12.9 | FAM83A | IGFALS | MEP1B | PF4 | RP11-169F17.1 | SERP2 | TMEM55A |
| AC046143.7 | C11orf86 | CTC-459F4.1 | FAM85B | IGFBP1 | MESP1 | PFN2 | RP11-190J1.3 | SERTAD4 | TMEM61 |
| AC069277.2 | C12orf56 | CTC-459F4.3 | FAM86B1 | IGSF23 | MESP2 | PGM5P2 | RP11-197K6.1 | SEZ6 | TMEM65 |
| AC092653.5 | C16orf74 | CTD-2105E13.6 | FAM90A1 | INHA | MFAP3L | PI15 | RP11-211C9.1 | SGK110 | TNFRSF11B |
| AC092669.3 | C19orf69 | CTD-2377D24.4 | FAXC | INSM1 | MFSD6L | PIFO | RP11-215A19.1 | SGSM1 | TNNC1 |
| AC097468.4 | C1orf115 | CTD-2377D24.6 | FBLL1 | ITLN1 | MIB2 | PITX2 | RP11-218M22.1 | SHISA2 | TNNC2 |
| AC099522.1 | C1orf51 | CTD-2554C21.3 | FBP2 | JPH3 | MLF1 | PIWIL1 | RP11-269C23.3 | SHISA3 | TPBG |
| AC112229.4 | C1QTNF3 | CTD-3060P21.1 | FBXL2 | KB-1247B1.1 | MLLT4 | PKIA | RP11-274H2.5 | SI | TPD52L1 |
| AC124789.1 | C2CD4B | CTF1 | FBXO2 | KB-1460A1.5 | MLYCD | PLA2G12B | RP11-30J20.1 | SLC10A4 | TPM2 |
| AC131180.4 | C3orf67 | CTSF | FBXO27 | KBTBD12 | MME | PLA2R1 | RP11-30K9.5 | SLC13A2 | TREML2 |
| AC133785.1 | C4BPB | CXorf61 | FBXO44 | KCNB2 | MMP24 | PLAG1 | RP11-323F24.1 | SLC16A10 | TRIM54 |
| AC138430.4 | C4orf48 | CYorf17 | FEV | KCNF1 | MOCS1 | PLIN2 | RP11-347C12.3 | SLC16A14 | TRPC7 |
| AC140481.1 | C5orf42 | CYP2W1 | FKBP10 | KCNG1 | MOK | PLOD2 | RP11-353N14.2 | SLC16A9 | TRPC7 |
| AC140481.7 | C6orf123 | CYP4F2 | FNDC4 | KCNG3 | MRVI1 | PNMA6C | RP11-366F6.2 | SLC1A7 | TRPM6 |
| AC144521.1 | C7orf41 | CYP4F30P | FOXD1 | KCNH8 | MS4A15 | PNMT | RP11-368L12.1 | SLC22A3 | TSPY26P |
| AC145124.2 | C7orf57 | CYP4F31P | FOXL2 | KCNIP3 | MSI1 | PNPLA3 | RP11-371I1.2 | SLC25A21 | TTC40 |
| AC226118.1 | C8G | DACH1 | FOXP2 | KCNJ12 | MST1P2 | PON3 | RP11-379F4.4 | SLC29A4 | TTLL2 |
| ACAA2 | C9orf24 | DACT2 | FRMD1 | KCNK15 | MT1A | POPDC3 | RP11-384O8.1 | SLC30A10 | TTLL7 |
| ACHE | CA1 | DBN1 | FSCN1 | KCNK6 | MT1H | POTEF | RP11-385J1.2 | SLC35D3 | TUBAL3 |
| ADIRF | CA7 | DENND2C | FSTL5 | KCNK9 | MTNR1A | POTEI | RP11-386G11.5 | SLC36A4 | TUBB4A |
| ADM | CABP4 | DEPDC7 | FTCD | KCNMB3 | MUC12 | POU6F2 | RP11-386M24.6 | SLC38A4 | TUBB6 |
| ADSSL1 | CABYR | DLX3 | FUOM | KCNQ4 | MUC2 | PPIC | RP11-395B7.2 | SLC3A1 | TXNRD3 |
| AGAP11 | CACNB4 | DLX6 | FZD9 | KCNS1 | MXRA7 | PPIE | RP11-395G23.3 | SLC46A1 | UBXN10 |
| AGGF1P1 | CACNG4 | DLX6 | G0S2 | KCP | MYADML2 | PPP1R14A | RP11-396C23.2 | SLC4A3 | UCHL1 |
| AKR1E2 | CADM2 | DMRTA2 | GABRQ | KHDRBS3 | MYO18B | PPP1R14D | RP11-397A16.1 | SLC51B | ULBP2 |
| AL022344.7 | CADPS | DPP4 | GABRR1 | KIAA1257 | MYO3A | PPP1R1B | RP11-404P21.1 | SLC5A12 | ULK2 |
| AL050303.1 | CAMK2B | DRC1 | GALNT14 | KIAA1377 | MYO7B | PPP1R3C | RP11-408H20.1 | SLC6A10P | UPK1B |
| ALG1L | CAMKK1 | DRD2 | GALNT15 | KIAA1644 | MYOM3 | PPP1R3G | RP11-425M5.5 | SLC6A20 | UPK3A |
| ALOX12P2 | CAPN9 | DRGX | GALNT8 | KIF26A | NAALADL1 | PRAME | RP11-439E19.7 | SLC6A7 | VANGL2 |
| ALPI | CAPS | DSCR4 | GDPD2 | KLF7 | NAGS | PRAP1 | RP11-462G2.1 | SLC6A8 | VWDE |
| ANGPTL4 | CASQ1 | DSCR8 | GFRA3 | KLHL35 | NANOS3 | PRKAA2 | RP11-475N22.4 | SLC7A4 | WASF3 |
| ANKRD33 | CAV2 | DTNA | GGACT | KLK1 | NAT2 | PRKAG2 | RP11-529A4.4 | SLC7A9 | WASIR2 |
| ANPEP | CCL14 | DUOX1 | GJB5 | KLK10 | NAT8 | PRKCDBP | RP11-537P22.1 | SMC1B | WDR72 |
| AP000769.1 | CCNJL | DUOX2 | GNAI1 | KLK12 | NAT8L | PRLR | RP11-539I5.1 | SMIM1 | WIPF3 |
| AP001065.15 | CD109 | DUOXA2 | GNG4 | KLK13 | NDNF | PROC | RP11-540A21.2 | SNAP25 | WISP2 |
| AP001065.2 | CDH17 | DUSP23 | GNGT1 | KLRG2 | NDP | PRPH | RP11-559N14.5 | SNTA1 | WISP3 |
| AP001631.9 | CDHR2 | DUSP9 | GPR158 | KRT7 | NECAB1 | PRSS21 | RP11-575F12.2 | SOHLH2 | WNK2 |
| AP005482.1 | CDIP1 | ECHDC2 | GPR37L1 | LANCL3 | NETO2 | PRSS51 | RP11-59E19.1 | SOWAHA | WNK4 |
| APLP1 | CDX1 | EEF1A2 | GPR63 | LARP6 | NEU4 | PTGS2 | RP11-5P18.10 | SPDYC | WNT11 |
| APOA1 | CDX2 | EGFR | GREB1 | LDLRAD3 | NEURL2 | PTPRN | RP11-61A14.1 | SPEF2 | WNT16 |
| APOB | CELF4 | EGR4 | GREB1L | LINC00085 | NHLRC1 | PYGO1 | RP11-642C5.1 | SRPK3 | WNT7B |
| APOC3 | CELP | EIF3FP1 | GRIA3 | LINC00086 | NINL | QPRT | RP11-64C12.3 | SRPX | XPNPEP2 |
| AQP12B | CES4A | ELOVL4 | GRID2 | LINC00162 | NKD2 | RAB34 | RP11-697M17.1 | SST | YBX2 |
| AQPEP | CHADL | EN2 | GRIP1 | LINC00346 | NKX2-5 | RAB36 | RP1-170O19.20 | SSUH2 | ZG16 |
| ARC | CHGA | ENTPD8 | GS1-120K12.4 | LINC00482 | NMB | RAB3B | RP11-74E22.4 | STAC | ZIC5 |
| ARHGAP31 | CHGB | EPHX3 | GUCY2C | LINC00535 | NMNAT2 | RAB6B | RP11-758M4.4 | STK32C | ZNF114 |
| ARHGDIG | CHP2 | EPHX4 | H2AFY2 | LINC00605 | NOL3 | RAC3 | RP11-760D2.11 | STMN3 | ZNF185 |
| ARHGEF4 | CHST5 | EREG | HCG11 | LINC00654 | NOVA1 | RANBP17 | RP11-776H12.1 | SULT1A2 | ZNF385C |
| ASCL2 | CHST6 | ERVMER34-1 | HCN2 | LINC00662 | NPM2 | RASGEF1A | RP11-78F17.1 | SULT4A1 | ZNF470 |
| ATP2B3 | CIB2 | ESPN | HEPH | LINC00668 | NPPC | RASSF8 | RP11-794G24.1 | SYCE1L | ZNF503 |
| ATP6V0E2 | CISD1 | ESPNL | HMGCS2 | LINC00857 | NPW | RBP4 | RP11-797H7.5 | SYN3 | ZNF530 |
| ATP6V1C2 | CITED1 | ESPNP | HOMER2 | LINC00865 | NRIP3 | RCOR2 | RP11-89K21.1 | SYT1 | ZNF718 |
| ATRNL1 | CKB | ESYT3 | HOXA | LIPC | NSMF | REEP6 | RP11-933H2.4 | SYT7 | ZNF876P |
| AXDND1 | CKMT1B | ETNK2 | HOXA | LPPR1 | NTF3 | RET | RP11-981G7.2 | TBX10 | ZSCAN31 |
