# Supplementary material for: Interaction of Epstein-Barr virus genes with human gastric carcinoma transcriptome
Source: Oncotarget. 2017 Mar 21;8(24):38399–412. doi: 10.18632/oncotarget.16417 (PMC5503541; doi:10.18632/oncotarget.16417)
Supplement: Supplementary file 4 [file oncotarget-08-38399-s004.docx]

**Table 11: Selected EBV+ and EBV− samples**

| EBV+ sample | Histological diagnosis of EBV+ sample | Selected EBV− sample | Histological diagnosis of EBV− sample | Distance to the selected EBV− sample | Average distance to all the 260 EBV− sample |
| --- | --- | --- | --- | --- | --- |
| TCGA-B7-5818_EBV | Stomach Adenocarcinoma Diffuse Type | TCGA-D7-6522 | Stomach Adenocarcinoma Diffuse Type | 0.032 | 0.243 |
| TCGA-BR-4253_EBV | Stomach Adenocarcinoma NOS | TCGA-BR-4255 | Stomach Adenocarcinoma NOS | 0.008 | 0.228 |
| TCGA-BR-6455_EBV | Stomach Adenocarcinoma NOS | TCGA-BR-6457 | Stomach Adenocarcinoma NOS | 0.045 | 0.199 |
| TCGA-BR-6706_EBV | Stomach Adenocarcinoma NOS | TCGA-BR-A4QM | Stomach Adenocarcinoma NOS | 0.020 | 0.205 |
| TCGA-BR-6707_EBV | Stomach Adenocarcinoma NOS | TCGA-BR-6457 | Stomach Adenocarcinoma NOS | 0.011 | 0.209 |
| TCGA-BR-7196_EBV | Stomach Adenocarcinoma NOS | TCGA-BR-8371 | Stomach Adenocarcinoma NOS | 0.074 | 0.285 |
| TCGA-BR-7958_EBV | Stomach Adenocarcinoma NOS | TCGA-BR-8590 | Stomach Adenocarcinoma NOS | 0.047 | 0.225 |
| TCGA-BR-8366_EBV | Stomach Adenocarcinoma NOS | TCGA-BR-6452 | Stomach Adenocarcinoma NOS | 0.004 | 0.237 |
| TCGA-BR-8381_EBV | Stomach Adenocarcinoma NOS | TCGA-BR-6453 | Stomach Adenocarcinoma NOS | 0.045 | 0.210 |
| TCGA-BR-8589_EBV | Stomach Adenocarcinoma NOS | TCGA-BR-8590 | Stomach Adenocarcinoma NOS | 0.037 | 0.223 |
| TCGA-BR-8676_EBV | Stomach Intestinal Adenocarcinoma NOS | TCGA-F1-A448 | Stomach Intestinal Adenocarcinoma Mucinous Type | 0.053 | 0.247 |
| TCGA-BR-8686_EBV | Stomach Intestinal Adenocarcinoma Tubular Type | TCGA-BR-8683 | Stomach Intestinal Adenocarcinoma Tubular Type | 0.037 | 0.234 |
| TCGA-BR-A4J4_EBV | Stomach Adenocarcinoma NOS | TCGA-BR-A4IV | Stomach Adenocarcinoma Diffuse Type | 0.031 | 0.256 |
| TCGA-CD-5801_EBV | Stomach Adenocarcinoma NOS | TCGA-BR-A4J1 | Stomach Adenocarcinoma NOS | 0.031 | 0.205 |
| TCGA-CG-5722_EBV | Stomach Intestinal Adenocarcinoma NOS | TCGA-CG-4440 | Stomach Intestinal Adenocarcinoma NOS | 0.021 | 0.317 |
| TCGA-D7-5577_EBV | Stomach Intestinal Adenocarcinoma Tubular Type | TCGA-BR-8690 | Stomach Adenocarcinoma NOS | 0.055 | 0.271 |
| TCGA-D7-8570_EBV | Stomach Intestinal Adenocarcinoma Tubular Type | TCGA-BR-8371 | Stomach Adenocarcinoma NOS | 0.049 | 0.263 |
| TCGA-D7-8573_EBV | Stomach Intestinal Adenocarcinoma Tubular Type | TCGA-BR-6457 | Stomach Adenocarcinoma NOS | 0.038 | 0.218 |
| TCGA-D7-A4YX_EBV | Stomach Adenocarcinoma NOS | TCGA-BR-6457 | Stomach Adenocarcinoma NOS | 0.037 | 0.200 |
| TCGA-FP-7916_EBV | Stomach Adenocarcinoma Diffuse Type | TCGA-CG-5724 | Stomach Adenocarcinoma Diffuse Type | 0.143 | 0.351 |
| TCGA-FP-7998_EBV | Stomach Adenocarcinoma Diffuse Type | TCGA-BR-A4CS | Stomach Adenocarcinoma NOS | 0.127 | 0.342 |
| TCGA-HU-8608_EBV | Stomach Adenocarcinoma Diffuse Type | TCGA-HU-A4GC | Stomach Adenocarcinoma Diffuse Type | 0.032 | 0.242 |
| TCGA-HU-A4G2_EBV | Stomach Adenocarcinoma Diffuse Type | TCGA-CD-A487 | Stomach Adenocarcinoma Diffuse Type | 0.011 | 0.255 |
| TCGA-HU-A4G6_EBV | Stomach Intestinal Adenocarcinoma Papillary Type | TCGA-CG-5725 | Stomach Adenocarcinoma NOS | 0.063 | 0.293 |
| TCGA-HU-A4H0_EBV | Stomach Adenocarcinoma Diffuse Type | TCGA-HU-8243 | Stomach Intestinal Adenocarcinoma Tubular Type | 0.077 | 0.284 |

*Note:*

1. *Since some EBV+ samples share one EBV- sample with the closest distance, the number of selected EBV- samples for a total of 25 EBV+ samples is only 20.*
2. *NOS: Not otherwise specified*
